# Voltage-Dependent Anion Selective Channel 3: Unraveling Structural and Functional Features of the Least Known Porin Isoform

**DOI:** 10.3389/fphys.2021.784867

**Published:** 2022-01-10

**Authors:** Simona Reina, Vanessa Checchetto

**Affiliations:** ^1^Department of Biomedical and Biotechnological Sciences, University of Catania, Catania, Italy; ^2^Department of Biology, University of Padova, Padua, Italy

**Keywords:** VDAC3, electrophysiology, planar lipid bilayer, redox signaling, human pathologies

## Abstract

Voltage-dependent anion-selective channels (VDAC) are pore-forming proteins located in the outer mitochondrial membrane. Three isoforms are encoded by separate genes in mammals (VDAC1-3). These proteins play a crucial role in the cell, forming the primary interface between mitochondrial and cellular metabolisms. Research on the role of VDACs in the cell is a rapidly growing field, but the function of VDAC3 remains elusive. The high-sequence similarity between isoforms suggests a similar pore-forming structure. Electrophysiological analyzes revealed that VDAC3 works as a channel; however, its gating and regulation remain debated. A comparison between VDAC3 and VDAC1-2 underlines the presence of a higher number of cysteines in both isoforms 2 and 3. Recent mass spectrometry data demonstrated that the redox state of VDAC3 cysteines is evolutionarily conserved. Accordingly, these residues were always detected as totally reduced or partially oxidized, thus susceptible to disulfide exchange. The deletion of selected cysteines significantly influences the function of the channel. Some cysteine mutants of VDAC3 exhibited distinct kinetic behavior, conductance values and voltage dependence, suggesting that channel activity can be modulated by cysteine reduction/oxidation. These properties point to VDAC3 as a possible marker of redox signaling in the mitochondrial intermembrane space. Here, we summarize our current knowledge about VDAC3 predicted structure, physiological role and regulation, and possible future directions in this research field.

## Introduction

The Voltage-dependent anion-selective channels (VDACs) are pore-forming proteins, also known as porins, localized in the mitochondrial outer membrane (MOM). These small proteins (30–35 kDa) are the main pathway for the flux of ions and metabolites between mitochondria and the cytoplasm. VDACs are involved in many cellular functions, including Adenosine DiPhosphate and Adenosine TriPhosphate transfer, Reactive Oxygen Species (ROS) signaling, hexokinase anchoring, and apoptosis (Vander Heiden et al., 2000). In mammals and most chordates, three VDAC isoforms have been characterized: VDAC1, VDAC2, and VDAC3 (Ha et al., 1993; Sampson et al., 1996, 1998; Messina et al., 2012).

The data emerging in the last decades denote that VDAC isoforms in mammals show differences in (i) the mitochondrial localization: VDAC1 and VDAC2 are colocalized within the same restricted area in the MOM, while VDAC3 is widely distributed on the MOM (Neumann et al., 2010; Okazaki et al., 2015); (ii) the channel activity and voltage dependence: both VDAC1 and VDAC2 are maximally open at 0 mV and that they enter a lower-conductance state. They work mainly as anion channels in the −40 a +40 mV voltage range, while outside of this range, they function as cation channels. On the contrary, VDAC3 did not exhibit typical voltage gating and electrophysiological properties (Checchetto et al., 2014; Okazaki et al., 2015); and (iii) the N-terminal sequence and its contribution to cell viability and survival: the N-terminal end of VDACs contains amphipathic α-helix elements with functionally relevant properties (Ujwal et al., 2008). A remarkable difference in the number of cysteines in the VDAC N-terminal sequences, VDAC3 has two cysteines at positions 2 and 8, VDAC2 also has two cysteines, but only one of them corresponding to the VDAC3 In addition, VDAC1 and 2 N-termini have additional residues, the target of carbonylation reactions, while VDAC3 does not have them; and (iv) finally the specific Protein-Protein Interactions (PPIs). These data lead to hypothesize a more specialized role for each isoform in different biological contexts. The PPIs of VDAC1 have been described more in-depth than those of VDAC2 and VDAC3 (Caterino et al., 2017).

**FIGURE 1:**
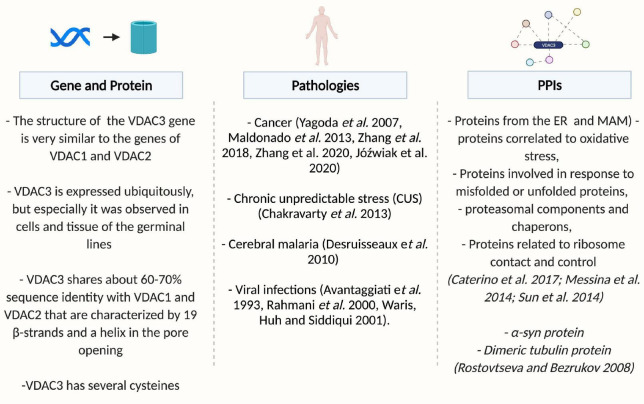
Overview of VDAC3. Created by BioRender.com.

This review will report progress in understanding the VDAC3 function, focusing on its structure, and discussing various models proposed for voltage gating, its modulation, and its overall role as a channel (see Figure 1).

## Overview of Voltage-Dependent Anion Selective Channel 3: From Gene to Protein Structure

In higher eukaryotes, the structure of VDAC genes is very similar. The genes have the same coding-exon organization, the same size, with the VDAC2 gene containing an additional first exon encoding for the short presequence of 11 amino acids, a feature of this isoform. The size of the VDACs introns varies, but the exon-intron organization is conserved among the whole group (Young et al., 2007). Evolutionary analysis indicates that VDAC3 is the oldest of the vertebrate VDAC genes, suggesting that multiple isoforms arose from gene duplication and VDAC3 diverged from the primordial VDAC first, with VDAC1 and VDAC2 arising more recently. VDAC3 is placed on a separate branch of a phylogenetic tree, suggesting that this isoform has a distinctive physiological function from VDAC1 and VDAC2 (Sampson et al., 1996). The observation indirectly supports this prediction that VDAC3 does not rescue the porin-less yeast temperature-sensitive phenotype completely but generates a lower level of growth under restrictive conditions (Sampson et al., 1997).

Transcription of the VDAC isoform genes was detected with many techniques and indicated that the three mammalian VDAC isoforms are ubiquitously expressed. To date, very little is known about the mechanisms of VDAC3 gene regulation. Although its transcript is less abundant within the cell, the *VDAC3* promoter exhibits the highest transcriptional activity compared to VDAC2 and, particularly, VDAC1. In this regard, it has been hypothesized that VDAC3 transcripts could be less stable than VDAC1 ones or that their levels are kept constitutively high to readily increase VDAC3 expression in response to specific stimuli (Zinghirino et al., 2020). Consequently, the VDAC3 promoter contains a polypyrimidine stretch that has been featured as a specific target of oxidative stress (Nepal et al., 2020).

Former studies based on structure prediction suggested that VDAC isoforms folded similarly to bacterial porins (Mihara and Sato, 1985; Kleene et al., 1987; Young et al., 2007). About 20 years after the primary structure elucidation, the VDAC1 3D structure was solved combining NMR spectroscopy and X-ray crystallography approaches in three different laboratories (Bayrhuber et al., 2008; Hiller et al., 2008; Ujwal et al., 2008), while the zfVDAC2 structure was solved by Schredelseker et al. (2014) and Gattin et al. (2015). Both VDAC1 from mice or humans and VDAC2 from zebrafish fold into a novel structure comprised of 19 β-strands and an N-terminal α-helix that adopted several conformations. A similar topology with 19 β-strands and a helix was recently resolved in TOM40, also located in the MOM (Araiso et al., 2019; Tucker and Park, 2019). VDAC3 shares about 60–70% sequence identity with VDAC1 and VDAC2. The sample preparation and spectroscopic methods described by Eddy et al. for VDAC2 will likely apply to this isoform as well (Eddy et al., 2019), but unfortunately, the 3D structure of VDAC3 has not yet been obtained, and only a few pieces of information are available so far. However, modeling of the hVDAC3 sequence on the structure of hVDAC1 or mVDAC1 showed a remarkable similarity, indicating that this isoform is possibly folded like the other two known VDACs.

Several investigations have focused on the N-terminal segment, which resides in the lumen and is not part of the VDACs barrel. The N-terminal region constitutes a mobile component of the protein that exhibits motion during voltage gating (Mannella et al., 1992; Mannella, 1997; Najbauer et al., 2021) and may modulate the interaction of the antiapoptotic proteins, HK and Bcl-2, to their binding sites (Shi et al., 2003; Abu-Hamad et al., 2009; Arzoine et al., 2009). The N-terminal sequence of VDACs is composed of 25 residues, except for VDAC2, where there is an N-terminal extension of 11 residues that do not change the channel activity. The VDAC isoforms also differ in their cysteine content. Human VDAC3 has six cysteines: four of these residues are predicted to be in the connection loops between β-strands, protruding toward the intermembrane space (IMS; Cys36, Cys65, Cys122, and Cys229), and two are in the N-terminal domain (Cys2 and Cys8). VDAC1 lacks cysteines at the N-terminus and has only two residues located on the β-strands (i.e., Cys 127 and Cys232 in humans). VDAC2 also has many cysteines (nine in humans and six in mice), but only one of them is conserved in the N-terminus in a position corresponding to VDAC3. The predicted location of hVDAC3 cysteines suggests that they are highly accessible to soluble oxidative molecules and related to some specific biological function.

To explore the role of the N-terminal domain of VDAC3, a set of chimerical proteins was created by swapping the first 20 amino acids of VDAC3 N-terminal with homologous sequences of the other isoforms and then expressing them in the yeast strain of *Saccharomyces cerevisiae* Δpor1 strain. Such replacement is sufficient to change the features of the protein radically. Insertion of the N-terminus of VDAC1 confers activity to VDAC3 and increases life span, indicating more efficient bioenergetic metabolism and/or better protection against ROS. However, also substitution with the N-terminus VDAC2 improves the ability of VDAC3 to complement the absence of endogenous porin1 in yeast, although to a lesser extent (Reina et al., 2010).

## Channel Activity of Voltage-Dependent Anion Selective Channel 3

The voltage-dependent characteristics of VDAC1 and VDAC2 have been extensively demonstrated, while those of VDAC3 has only recently been examined in detailed biophysical and electrophysiological studies (Table 1).

**TABLE 1:** Summary of information on VDAC3 channel activity.

Protein	mVDAC3 (Xu et al., 1999)	hVDAC3 (Checchetto et al., 2014)	hVDAC3 (Okazaki et al., 2015)	hVDAC3 (Queralt-Martín et al., 2020)	hVDAC3 (Conti Nibali et al., 2021)
Refolding detergent	5% DMSO, 2.5% Triton X-100	1% (v/v) LDAO	0,4% (v/v) LDAO	0,1% (v/v) LDAO	1% (v/v) LDAO
Reducing agent	The presence of DTT resulted in a modest increase in insertion of VDAC3 channels.	None	None	DTT	Without and with 5 mM DTT
Bilayer composition	Asolectin:cholesterol (5:1)	Asolectin (2 mg/ml)	POPE:POPC (8:2)	DOPC:DOPE:DOPG (1:1:2)	1% DiPhPC
Conductance	It does not show a clear preferred state for this channel in a phospholipid membrane.	∼90 pS in 1M KCl	∼500 pS in 250 mM KCl	∼3.9 nS in 1M KCl	In the absence of DTT ∼0.7 nS; in the presence of DTT ∼3 nS in 1M KCl
Voltage dependence	No	No	No	Yes	No A perfect overlap of the voltage dependence between all three isoforms was obtained only when the cysteines were removed from the hVDAC3 sequence
Ion selectivity	Similar to mVDAC1 and mVDAC2, mVDAC3 resulted in the same molecular weight cutoff, indicating that this protein could also form channels that allow the flux of large nonelectrolytes across the mem- brane.	N.D.	N.D.	Similar to hVDAC1	In the presence of DTT similar to hVDAC1 and hVDAC2 The results of these experiments further corroborate the importance of cysteine redox state in pore function, and they foster the hypothesis that the selectivity of the channel is correlated to the size of the unitary conductance.

Initially, Xu et al. (1999) demonstrated that mVDAC3 exhibited electrophysiological properties strikingly different from the other isoforms. mVDAC3 rarely insert into artificial membranes and did not gate well even at high membrane potentials (do not exhibit voltage-gating up to 80 mV; Xu et al., 1999). Subsequently, differences in the human isoform were also observed. As reported in Checchetto et al. (2014), the LDAO-solubilized hVDAC3 showed channel activity with very small conductance (90 pS in 1M KCl) compared to hVDAC1 conductance (>3,500 pS in 1M KCl), allowing passage of both chloride and gluconate anions. Unlike VDAC1, the VDAC3 channel was open even at transmembrane voltages higher than ±40 mV and showed a relatively high probability of opening even at ±80 mV. In addition, the pores were only slightly voltage-dependent and tended to adopt low conductance states preferentially at negative voltages lower than positive voltages (Checchetto et al., 2014). The small conductance matches the cellular performance of hVDAC3 expressed in yeast strain *S. cerevisiae* Δpor1 strain, where only partial growth recovery under non-permissive conditions (i.e., 2% glycerol at 37°C) was obtained (Xu et al., 1999; Reina et al., 2010; Checchetto et al., 2014; Okazaki et al., 2015). Careful analyzes showed that, after treatment with reducing agents, VDAC3 occasionally reaches the characteristic conductance level of a fully open VDAC (Okazaki et al., 2015; Reina et al., 2016). Very recently, the group of De Pinto further confirmed what was previously reported in Checchetto et al. (2014) using nanodisc-stabilized human VDACs: accordingly, in the absence of any reductants, VDAC3 inserted into artificial membranes as small, non-gated channels (Conti Nibali et al., 2021). When cysteines are found to be reduced in mass spectrometry analysis (following DTT and iodoacetamide treatment) it means that those cysteines were probably involved in disulfide bridges (otherwise DTT could not have reduced them back to SH). The main hypothesis for this discrepancy in hVDAC3 conductance was proposed to arise from the lipids used in planar lipid bilayer experiments and the difficulty in obtaining stable and homogeneous VDAC3 proteins. As reported in Queralt-Martín et al. (2020), VDAC3 forms canonical pores responsive to membrane voltage, even though with a much lower insertion rate than isoforms 1 and 2, and exclusively following highly-reducing refolding procedures. The authors correlated these differences with the lower stability of hVDAC3 in LDAO detergent. Using a highly reactive thiol-specific fluorochrome, they performed a thermal stability assay on mVDAC1 and hVDAC3, confirming a dramatic change in melting temperatures between the two isoforms (hVDAC3 *T*_*m*_ = 29°C, mVDAC1 *T*_*m*_ = 56°C). The outcome revealed that hVDAC3 has lower protein stability than hVDAC1 when solubilized in LDAO. The lower *T*_*m*_ value of hVDAC3 may explain the formation of noisy channels in the PLM due to the insertion of the unproperly folded hVDAC3. In this paper, the authors suggested that the best insertion yield is achieved using lipid bicelles made from 2-dimyristoyl-sn-glycero-3-phosphocholine (Queralt-Martín et al., 2020).

More often, the protein appears to form aggregates during protein purification. Another possible reason for such strange channel behavior of VDAC3 was attributed to the number and endomitochondrial location of exposed cysteine residues, which predominantly protrude toward IMS. According to mass spectrometry analysis, these amino acids follow an oxidative pattern that is conserved throughout evolution and does not include irreversible oxidations, as it was instead found in VDAC1 and VDAC2 cysteines (Saletti et al., 2018; Pittalà et al., 2020). In hVDAC3, Cys2, Cys8, Cys122, and Cys229 were identified as completely reduced, while Cys36 and Cys65 were detected in both the reduced and trioxidized form. The consequence is that the reduced Cys, even though it can be oxidized, is always reduced back to -SH and avoids being irreversibly oxidized. Then, they are candidates to be protected in a disulfide bridge, or their function is linked to their reduction, indicating that they are structurally and functionally crucial for the protein itself. The swapping experiments mentioned above have already suggested the importance of N-terminal cysteine residues in the pore activity of VDAC3 (Reina et al., 2010). Later, electrophysiological data reported in Okazaki et al. (2015) and Reina et al. (2016) confirmed the essential role of such sulfur-containing amino acids in channel gating, proposing that the N-terminal residues Cys2 and Cys8 could form transient disulfide bonds capable of modulating the pore diameter or changing the charges exposed on the protein surface (Amodeo et al., 2014; Guardiani et al., 2016) and therefore its conductance. The current flow through VDAC3 is dramatically reduced, compared to VDAC1 and VDAC2, under non-reducing conditions (i.e., ∼90 pS vs. ∼3.5 nS in 1M KCl, respectively).

A pivotal role for VDAC3 cysteines in modulating mitochondrial ROS has also been proposed (Reina et al., 2016). To date, however, this point is still speculation since no empirical evidence is available. In this regard, the latest data in the literature seem to support this hypothesis, at least indirectly: for instance, Zou et al. (2018) described the correlation between VDAC3 knockout and mitochondrial ROS overload in renal tubules in mice subjected to high salt intake. However, it is not clear how this “ROS buffering” activity should take place: one possible explanation is that under mitochondrial stress, conformational changes induced by –SO3H formation could function as signals for incorporation of VDAC3 into MDVs, responsible for the removal of oxidized proteins and closely involved in mitochondrial quality control (Soubannier et al., 2012; Reina et al., 2020). MDVs contain numerous oxidized proteins derived mainly from the MOM: VDAC1 has been listed among these proteins (Soubannier et al., 2012), whereas information on the presence of VDAC3 is not yet available.

To address their physiological role (Queralt-Martín et al., 2020) analyzed the activity of the hVDAC3 cysteine-less mutant (in which all six cysteine residues were replaced with alanines) and compared it with the WT form. The PLM results suggested that cysteine residues do not significantly affect the stability or functionality; however, they affect the ability of hVDAC3 to interact with other proteins (e.g., α-synuclein).

## Voltage-Dependent Anion Selective Channel 3-Protein Interactions

Protein interaction networks are crucial to understanding cell functions and pathways and developing successful therapies to treat human diseases.

In 2014, the VDAC3 interactome was defined *in vivo* by a TAP-Tag immunoprecipitation strategy and mass spectrometry identification (Messina et al., 2014). The crucial interactions correlate VDAC3 with: (i) proteins from the endoplasmic reticulum and MAM, (ii) proteins correlated with the response to oxidative stress, (iii) proteins involved in the response to misfolded or unfolded proteins, (iv) proteasomal components and chaperons, and (v) proteins related to ribosome contact and control (Messina et al., 2014).

In the context of PPIs, it has recently been reported that the main difference between VDAC3 and the other VDAC isoforms concerns associations with cytosolic proteins involved in mitochondrial metabolism, especially α-syn and the dimeric tubulin (Rostovtseva and Bezrukov, 2008). Several studies establish the involvement of α-syn in mitochondrial dysfunction. A detailed examination of the blockage kinetics of rVDAC1 reconstituted into planar lipid membranes defines that at nanomolar concentrations, α-syn reversibly causes time-resolved reversible blockages of the channel conductance. α-Syn induces two distinct blocked states, depending on its concentration and the applied voltage. Two steps characterize the blocked state in terms of conductance: a blocked state with a conductance of ∼40% that of the open state and a second deeper state with a conductance of ∼17% that of the open state (at potentials V ≥ 30 mV). α-Syn blocks rVDAC1 from both sides of the channel, but only when a negative potential is applied from the side of the α-syn addition, suggesting that the negatively charged C-terminal region of α-syn is responsible for the blockage of rVDAC1 (Rostovtseva et al., 2015). These recent data show that similarly to rVDAC1, α-syn interacts with VDAC3 but 10–100 times less effectively (Queralt-Martín et al., 2020). An important role is attributed to the VDAC3 cysteines. Queralt-Martin and colleagues, using a cysteine-less hVDAC3 mutant, showed that the cysteine residues do not significantly affect hVDAC3 stability or functionality, as previously indicated (De Pinto et al., 2016; Reina et al., 2016), but they are responsible for the voltage asymmetry in the on-rate of α-syn-hVDAC3 interaction (Queralt-Martín et al., 2020). Likewise, the authors reported that VDAC3 is blocked by tubulin 10 times less effective than isoform 1, supporting the hypothesis that VDAC3 is primarily open when VDAC1 is closed via tubulin interaction (Queralt-Martín et al., 2020).

Voltage-dependent anion selective channel 3 is involved in the recruitment of PINK1/Parkin, cytosolic proteins involved in a pathway regulating mitochondrial quality control and promoting the selective autophagy of depolarized mitochondria (mitophagy; Narendra et al., 2008, 2010; Geisler et al., 2010; Truban et al., 2017; Ge et al., 2020). Loss of its function causes profound morphological and functional alterations in mitochondria, associated with the pathogenesis of Parkinson’s disease. Sun et al. (2012) proposed that VDACs are part of the machinery that recruits Parkin to the organelle. Thus, they assumed that VDACs act as mitochondrial docking sites to recruit Parkin from the cytosol to mitochondria. The authors observed that in the absence of all three VDACs, the recruitment of Parkin to defective mitochondria and consequent mitophagy was compromised (Sun et al., 2012).

Another important VDAC-interactor is the VCP, a central and important element of the ubiquitin system. VCP is implicated in numerous neurodegenerative disorders. For example, its gene mutations cause frontotemporal dementia associated with inclusion body myopathy, early-onset Paget disease, familial ALS, and FTLD. Furthermore, VCP seems to act on VDAC3, addressing it toward microtubules through the traffic of cytoplasmic granules and enriching near the centrosome (Messina et al., 2014).

All these PPIs interactions are of great interest. They can have significant implications for mitochondrial bioenergetics and open the way to discover new possible specific *in vivo* functions of the VDAC3 isoform hitherto unexplored.

## Role of Voltage-Dependent Anion Selective Channel 3 in Pathologies

Due to its crucial role in cellular metabolism and apoptosis, VDAC proteins are implicated in a wide range of diseases (Caterino et al., 2017; Magrì et al., 2018), including cancer (Maldonado et al., 2010; Reina and De Pinto, 2017; Magrì et al., 2018), neurodegenerative disorders, including Parkinson’s disease (Rostovtseva et al., 2015), Amyotrophic Lateral Sclerosis (Magrì et al., 2016; Magri and Messina, 2017), and Alzheimer’s disease (Manczak and Reddy, 2012). The knowledge about VDAC3 involvement in pathologies is very restricted. Studies with erastin, a small molecule compound that selectively kills human tumor cells carrying the oncogenes HRAS, KRAS, or BRAF, raised the possible connection between VDAC3 and cancer (Yagoda et al., 2007; Maldonado et al., 2013). In particular, a role of isoform 3 in the hepatocarcinogenesis induced by HBV infection was proposed by Zhang et al. (2018, 2020): a specific miRNA (miR-3928v) was shown to directly target and down-regulate VDAC3 expression and to promote hepatocarcinoma malignancy, by a still unclarified molecular mechanism. Recently, Józwiak et al. (2020) reported a significant increase in VDAC3 expression in non-metastatic endometrial cancers compared to normal tissue^1^. However, alterations in VDAC isoform 3 mRNA levels have also been registered in pathologies different from cancer, such as chronic unpredictable stress (Chakravarty et al., 2013), cerebral malaria (Desruisseaux et al., 2010), and viral infections (Avantaggiati et al., 1993; Rahmani et al., 2000; Waris et al., 2001).

A peculiar expression of VDAC3 isoform was observed in cells and tissue in the germinal lines of different organisms. Although VDAC1 is predominantly located in cells of reproductive organs required for the development of gametes (Hinsch et al., 2001; Specchia et al., 2008), VDAC2 and VDAC3 are expressed in a specific portion of sperm and oocyte, and genetic variants or aberrant regulation of these genes are correlated with infertility (Sampson et al., 1997; Pan et al., 2017). VDAC3-deficient mice are healthy, but males are infertile with a disassembled sperm tail, the flagellum essential for sperm motility. In VDAC3-deficient mice, the normal structures found in spermatids within the testes suggest that the defect develops with the maturation of the sperm in the transition from the testes to the epididymis. Each microtubule doublet has a corresponding outer dense fiber, all of which are morphologically distinguishable. Two of the outer dense fibers that are associated with microtubules 3 and 8 terminate within the principal piece and form the longitudinal columns of the fibrous sheath that partition the axoneme into two unequal compartments (Sampson et al., 2001). The VDAC3 gene might affect the energy supply for spermatogenesis and Leydig cell steroidogenesis and, finally, affect spermatogenesis (Hinsch et al., 2004).

Although VDACs are highly conserved between species, the specific function of each isoform remains unknown. To understand the specialized biological role of VDACs isoforms, recently, using the main available public resource reporting high-throughput data of international collaborative projects (Zinghirino et al., 2020) was performed a systematic analysis of human VDAC gene promoters was performed to highlight their structural and functional features. In particular, the authors underlined that the most active promoter controls VDAC3, enriching in GC repetitions, suggesting an epigenetic control mechanism capable of reducing transcript expression. Factors binding sites found in the VDAC3 promoter belong to various families, but those involved in the development of germinal tissues, organogenesis, and sex determination are the most abundant, confirming the experimental evidence of its crucial role in fertility (Sampson et al., 2001).

## Future Perspectives and Conclusion

In general, the data available nowadays confirm that the VDAC3 function is still not fully discernible. In the beginning, VDAC isoforms were considered rescue vessels to make up for deficiencies in other abundant isoforms. Whether or not VDAC3 knockout or overexpression could alter the expression of other isoforms has been addressed by Craigen’s group (Wu et al., 1999): accordingly, mouse ES cells are the first mammalian VDAC to knock out a model in which a compensatory increase in VDAC1 expression was registered for VDAC2−/− and VDAC3−/−.

Several hints make the study of this protein a very intriguing and promising field for acquiring deeper basic knowledge and for the development of diagnostic and therapeutic approaches to a wealth of pathologies such as cancer, respiratory or reproductive system diseases, renal or dermatological diseases, some myopathies, frontotemporal dementia, and neurodegenerative disorders.

## Author Contributions

Both authors contributed to the writing of the manuscript.

## Conflict of Interest

The authors declare that the research was conducted in the absence of any commercial or financial relationships that could be construed as a potential conflict of interest.

## Publisher’s Note

All claims expressed in this article are solely those of the authors and do not necessarily represent those of their affiliated organizations, or those of the publisher, the editors and the reviewers. Any product that may be evaluated in this article, or claim that may be made by its manufacturer, is not guaranteed or endorsed by the publisher.

## Footnotes

1
https://www.proteinatlas.org/ENSG00000078668-VDAC3/tissue

## Abbreviations

ADP: Adenosine DiPhosphate
ALS: Amyotrophic Lateral Sclerosis
ATP: Adenosine TriPhosphate
Bcl2: B-cell lymphoma 2
BRAF: B-Raf and V-Raf murine sarcoma viral oncogene homolog
Cys: Cysteines
FTLD: FrontoTemporal Lobar Degeneration
HBV: hepatitis B virus
HK: hexokinase
HRAS: Harvey rat sarcoma virus
hVDAC: human Voltage-Dependent Anion-selective Channels
KCl: potassium chloride
KRAS: Kirsten rat sarcoma viral oncogene homolog
LDAO: lauryl dimethylamine oxide
MAMs: mitochondrial-associated membrane proteins
MDVs: mitochondrial-derived vesicles
MOM: mitochondrial outer membrane
mVDAC: mouse Voltage-Dependent Anion-selective Channels
PLM: Planar Lipid Membranes
PPIs: Protein-Protein Interactions
pS: pico Siemens
ROS: Reactive Oxygen Species
rVDAC: rat Voltage-Dependent Anion-selective Channels
SO3H: sulfonic acid
TAP-Tag: Tandem Affinity Purification Tag
Tm: melting temperature
VCP: Valosin-Containing Protein
VDAC: Voltage-dependent anion-selective channels
zfVDAC2: zebrafish Voltage-Dependent Anion-selective Channels
α-syn: α-synuclein
ES: embryonic stem.
